# Supplementary material for: An ACE2-blocking antibody confers broad neutralization and protection against Omicron and other SARS-CoV-2 variants of concern
Source: Sci Immunol. 2022 Apr 26:eabp9312. doi: 10.1126/sciimmunol.abp9312 (PMC9097884; doi:10.1126/sciimmunol.abp9312)
Supplement: Supplementary file 1 — Figs. S1 to S10 Tables S1 to S5 [file sciimmunol.abp9312_sm.pdf]

Science Immunology
AAAS

Supplementary Materials for

An ACE2-blocking antibody confers broad neutralization and protection against Omicron and other SARS-CoV-2 variants of concern

Wenjuan Du *et al.*

Corresponding author: Berend-Jan Bosch, b.j.bosch@uu.nl

DOI: 10.1126/sciimmunol.abp9312

The PDF file includes:

Figs. S1 to S10
Tables S1 to S5

Other Supplementary Material for this manuscript includes the following:

Table S6

Supplementary Materials for

An ACE2-blocking antibody confers broad neutralization and protection against Omicron and other SARS-CoV-2 variants-of-concern

Wenjuan Du, Daniel L. Hurdiss, Dubravka Drabek, Anna Z. Mykytyn, Franziska K. Kaiser, Mariana González-Hernandez, Diego Muñoz-Santos, Mart M. Lamers, Rien van Haperen, Wentao Li, Ieva Drulyte, Chunyan Wang, Isabel Sola, Federico Armando, Georg Beythien, Malgorzata Ciurkiewicz, Wolfgang Baumgärtner, Kate Guilfoyle, Tony Smits, Joline van der Lee, Frank J.M. van Kuppeveld, Geert van Amerongen, Bart L. Haagmans, Luis Enjuanes, Albert D.M.E. Osterhaus, Frank Grosveld, and Berend-Jan Bosch*

*Corresponding author. Email: b.j.bosch@uu.nl

This Word file includes:

Fig. S1. Mouse immunization scheme and neutralizing activity of hybridoma supernatants.

Fig. S2. 87G7 epitope binning and ACE2 binding inhibition.

Fig. S3. Cryo-EM data processing of SARS-CoV-2 S bound to 87G7 Fab.

Fig. S4. Structural comparison of WRAIR-2125 and 87G7.

Fig. S5. Location of VOC/VOI mutations relative to the COV2-2196 epitope.

Fig. S6. Representative images of SARS-CoV antigen immunohistochemistry in lungs of mice.

Fig. S7. Representative images of SARS-CoV-2 induced histopathological lesions in the nasal turbinates of hamsters.

Fig. S8. Representative images of SARS-CoV antigen immunohistochemistry in nasal turbinates of hamsters.

Fig. S9. Representative images of SARS-CoV-2 induced histopathological lesions in the lung of hamsters.

Fig. S10. Representative images of SARS-CoV antigen immunohistochemistry in lungs of hamsters.

Table S1. Summary of data acquisition and image processing statistics.

Table S2. Scoring system of mice lung SARS-CoV antigen immunohistochemistry.

Table S3. Scoring system of hamster lung (alveolar, airway and vascular) lesions.

Table S4. Scoring system of nasal cavity lesions.

Table S5. Scoring system of hamster lung and nose SARS-CoV antigen immunohistochemistry

Table S6. Raw data file.

A

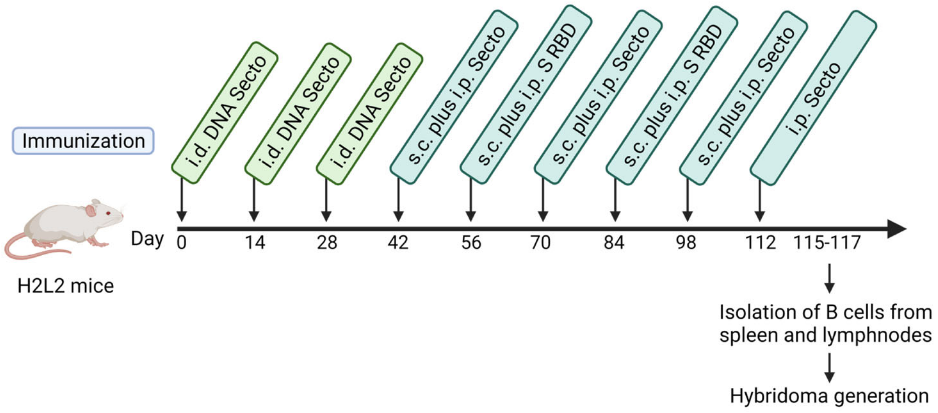

B

Hybridoma	Neutralization (%)	Hybridoma	Neutralization (%)	Hybridoma	Neutralization (%)	Hybridoma	Neutralization (%)	Hybridoma	Neutralization (%)	Hybridoma	Neutralization (%)
87g7	98	30a9	58	89g10	45	25c4	30	77h1	18	18b4	-4
88g10	93	22e10	58	87g3	45	77a10	30	22d12	18	27d12	-4
85a10	92	31b5	57	77c9	44	20d7	30	24c4	18	77f2	-6
86e6	92	29h1	57	24c3	43	69e5	30	17f5	18	59b10	-6
90c6	90	23g6	56	78d8	43	29g1	30	10e1	18	53a5	-7
85f8	89	36b8	56	23e10	43	30a11	29	57g2	17	15f12	-7
85h3	89	89a6	56	24b6	43	48d2	29	17h1	17	77d9	-8
86d7	87	22a12	55	90b6	42	83a12	29	45b2	17	19c7	-9
21e1	86	29a5	55	24b1	41	84d4	29	5a12	16	18a6	-9
83e6	86	85e2	55	20d4	41	39c4	29	18e2	15	76g6	-10
90a1	85	17f12	54	20c4	40	89c12	28	35d11	14	10g12	-10
80a7	83	27g1	54	20g7	40	4h12	28	76a11	14	10d11	-12
1h4	82	22c6	54	20e8	40	23g11	28	19g1	13	27a9	-12
86b7	81	22d11	53	22b10	40	21c4	27	23c8	13	19c1	-14
21g1	80	20e7	53	36g1	39	22a4	27	19b1	13	18h9	-14
21h2	75	38g11	52	49h7	39	20c10	27	1d5	13	47g2	-14
24e2	75	22d10	52	20a10	39	3e1	27	66a7	12	14h1	-15
19b4	73	15h5	52	53g7	38	84f4	27	23b12	11	24h5	-16
19g9	72	70h8	52	80a4	37	28f1	26	51c12	11	50d4	-17
25e7	70	29d5	51	81c5	37	25f10	26	23d11	11	30a2	-18
22f3	70	27g10	51	9a7	36	26e3	26	23g12	11	16f3	-20
83g1	70	88f5	51	15h10	36	23e11	25	9a4	11	28a7	-31
79a5	69	86e9	50	22d5	36	22a10	25	25e12	10	23d8	-33
30b8	69	22c3	50	26a2	36	25f5	25	25d5	10	76h8	-36
28g11	69	79c2	50	30h6	36	54f10	24	16a7	10	53a8	-37
24f1	67	41g6	49	25b7	36	76d2	24	48b9	9	45a9	-39
20g1	66	49h10	49	58h3	35	76b7	24	21a2	9	30d2	-40
24f2	66	23f6	49	10d2	35	23h10	23	22h1	8	24c5	-50
29a8	66	87b12	49	83b10	35	28d1	23	27g9	8	55a6	-51
5f5	65	44a2	48	82d5	35	27d4	23	24g6	7	46e3	-54
21b7	65	22e3	48	18d8	34	24a7	23	23b11	6	28e6	-59
20d12	65	25g1	48	40c11	34	42d11	22	55a5	5	7f11	-70
20a6	65	28d3	48	61h6	34	24b3	22	9e1	4	75e4	-72
88f1	65	85c1	47	28e3	33	9d4	22	16b11	2	52e10	-76
20b2	65	33g11	47	88d3	33	25g9	22	36a7	2	54h8	-76
22f7	64	37c12	47	84c7	32	25h2	22	49c2	2	12g4	-89
21h11	64	74b5	47	24b5	32	46d12	22	52e2	1	17b11	-91
29f4	64	25h9	47	30d1	32	15e7	21	22g5	1	16h10	-97
84b8	63	18b3	47	20c2	32	29c8	21	17e9	0	28b7	-98
44d10	61	22h8	46	23h1	31	38d8	20	77f7	0	53b10	-98
89a1	61	17h6	46	56f1	31	22a1	20	19c5	0	17h11	-102
77a2	61	89b3	46	42g5	31	42a10	20	21c3	0	56d9	-125
27a2	60	38h2	46	17e12	31	25c3	19	28e1	-1		
30c8	60	18h6	46	29g6	31	54a9	19	87b1	-1		
43c2	59	24a4	45	39b10	31	1f4	19	51a5	-2		
20c12	59	84e1	45	21h9	31	24a12	19	27f2	-2		

Figure S1. Mouse immunization scheme and neutralizing activity of hybridoma supernatants. (A) Mouse immunization scheme. H2L2 mice were immunized with plasmid DNA encoding the trimeric SARS-CoV-2 S ectodomain by intradermal electroporation (i.d.; 3x), followed by alternating protein immunizations with the ectodomain trimer (Secto) or receptor binding domain (RBD) of the SARS-CoV-2 S protein administered subcutaneously (s.c.) and/or intraperitoneally (i.p.) as indicated. **(B)** Screening of hybridoma supernatants for pseudovirus neutralization activity. Pseudovirus carrying the SARS-CoV-2 S^{E484K} protein

was preincubated with 1/10 volume of H2L2 hybridoma culture supernatant for 1h, prior to infection of Vero cells. The per cent neutralization is shown for each hybridoma supernatant. Hybridoma supernatants are ordered by neutralization potency, and red-to-white coloring denotes potent to no neutralization, respectively.

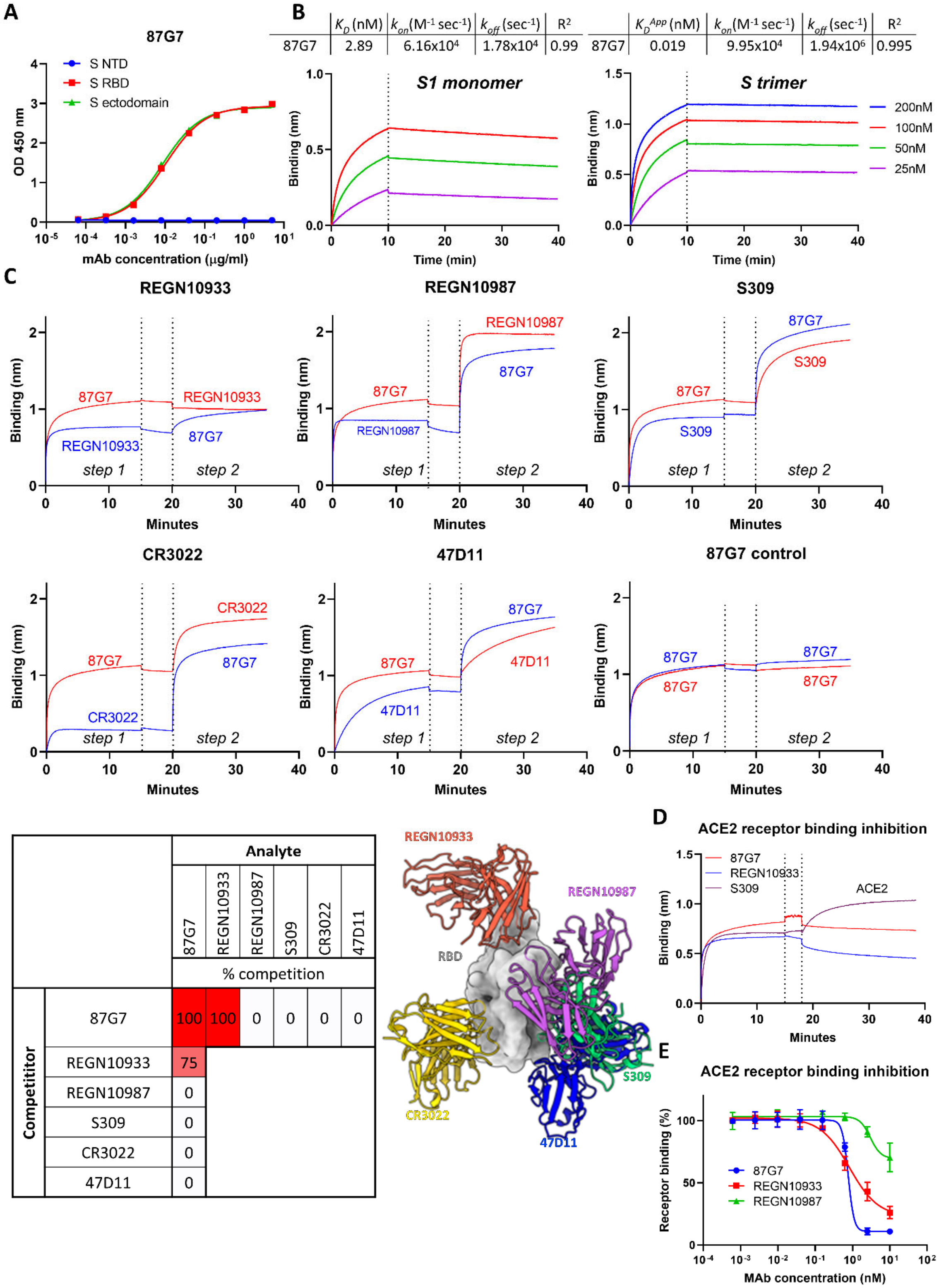

	K_D (nM)	k_{on} (M^{-1} sec^{-1})	k_{off} (sec^{-1})	R^2
87G7	2.89	6.16x10^4	1.78x10^4	0.99

	K_D^{App} (nM)	k_{on} (M^{-1} sec^{-1})	k_{off} (sec^{-1})	R^2
87G7	0.019	9.95x10^4	1.94x10^6	0.995

		Analyte					
		87G7	REGN10933	REGN10987	S309	CR3022	47D11
		% competition					
Competitor	87G7	100	100	0	0	0	0
	REGN10933	75					
	REGN10987	0					
	S309	0					
	CR3022	0					
	47D11	0					

Figure S2. 87G7 epitope binning and ACE2 binding inhibition. (A) ELISA binding curves of 87G7 to the plate-immobilized N-terminal domain (NTD), the receptor-binding domain (RBD) or ectodomain of SARS-CoV-2 S. **(B)** Binding kinetics of 87G7 to SARS-CoV-2 S measured by biolayer interferometry (BLI). 87G7 mAb was loaded at optimal concentration (21 nM) onto the anti-human Fc biosensor for 10 mins, after which association of antigen was achieved by incubating the sensor with a 2-fold dilution series of recombinant SARS-CoV-2 S1 monomer or S ectodomain trimer for 10 min, followed by a dissociation step in PBS for 30 min. K_D : equilibrium dissociation constant. k_{on} : association rate constant, k_{off} : dissociation rate constant. K_D^{App} reflects the 'apparent affinity' between IgG antibodies and spike trimer. **(C)** Binding competition between 87G7 and benchmarking antibodies to SARS-CoV-2 S ectodomain trimer evaluated using BLI. The strep-tagged SARS-CoV-2 S antigen was loaded to the anti-human Fc biosensor bound with antibodies against Strep tag. The competitor antibody was bound to spike (step 1) before incubation with the analyte antibody (step 2) as indicated, and percent competition bins are indicated in the table (dark red: >90% competition, light red; 40-80% competition, white: <10% competition). Data from a representative experiment (out of two) are shown. Benchmarking antibodies tested for binding competition with 87G7 are shown in complex with SARS-CoV-2 RBD and include REGN10933 (PDB: 6XDG), REGN10987 (PDB: 6XDG), S309 (parent of VIR-7831, PDB: 6WPS), CR3022 (PDB: 6W41) and 47D11 (PDB: 7AKD) **(D)** BLI-based receptor-binding inhibition assay. SARS-CoV-2 S ectodomain bound to the sensor was pre-incubated with 87G7 or two control mAbs REGN10933 (ACE2 binding competitor) or S309 (non-ACE2 competing), followed by a washing step and subsequent exposure to soluble human ACE2 receptor. The experiment was performed twice, data from a representative experiment is shown. **(E)** ELISA-based receptor-binding inhibition assay. SARS-CoV-2 S ectodomain pre-incubated with serially diluted 87G7 or two control mAbs REGN10933 or REGN10987 (both ACE2 binding competitors) was added to ELISA plates coated with soluble human ACE2. Spike binding to ACE2 was detected using an HRP-conjugated antibody recognizing the C-terminal Strep-tag on SARS-CoV-2 S ectodomain. Data points represent the average ± SDM, for n = 3 replicates from two independent experiments.

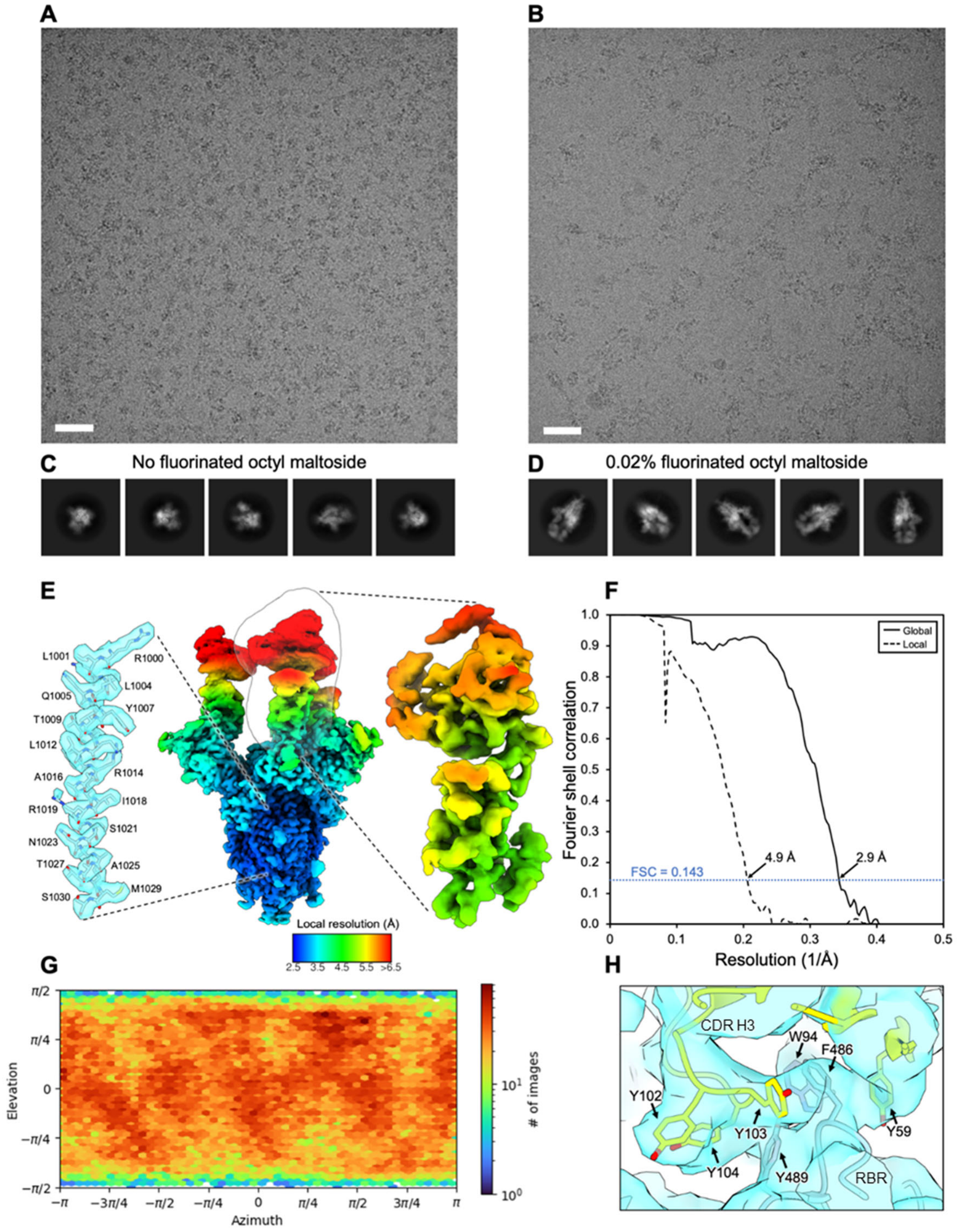

Figure S3. Cryo-EM data processing of SARS-CoV-2 S bound to 87G7 FAb (A) Representative motion-corrected micrograph of the 87G7-bound SARS-CoV-2 spike ectodomains embedded in vitreous ice. Scale bar = 50 nm. **(B)** As shown in A, for the sample incubated with 0.2% fluorinated octyl maltoside. **(C)** Representative reference-free 2D class averages generated in cryoSPARC. **(D)** As shown in C, for the sample incubated with 0.2% fluorinated octyl maltoside. **(E)** DeepEMhancer filtered EM density maps for the globally refined spike-87G7 complex and locally refined RBD-87G7 complex, colored according to local resolution which was calculated in Relion3.1. The outline of the local refinement mask is overlaid with the globally refined map. Representative density and fitted atomic coordinates for the S2 region of the globally refined map is shown on the left. **(F)** Gold-standard Fourier shell correlation (FSC) curves generated from the independent half maps contributing to the 2.9 Å resolution global refinement and 4.9 Å resolution local refinement. **(G)** Angular distribution calculated in cryoSPARC for particle projections in the local refinement. **(H)** Cryo-EM density for the locally refined 87G7 epitope-paratope region with the fitted atomic coordinates. RBD residues are colored blue and the light- and heavy-chain variable domains are colored purple and yellow, respectively.

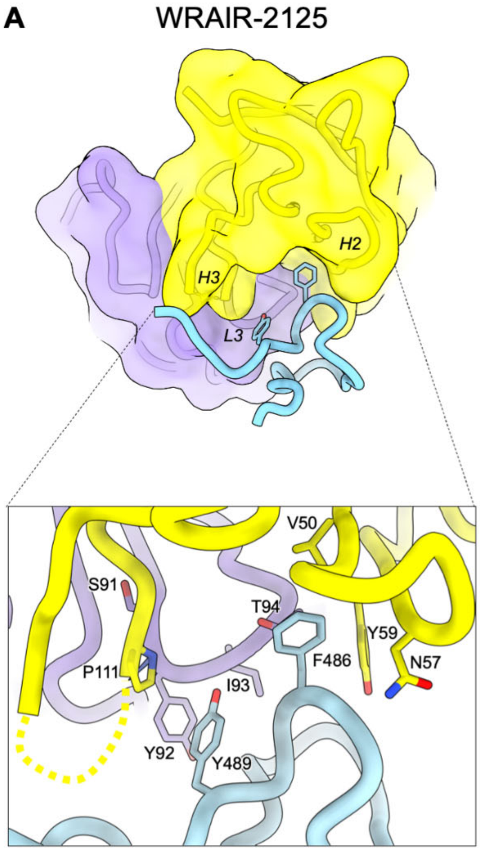

Figure S4. Structural comparison of WRAIR-2125 and 87G7. (A) The top panel shows a surface representation of the WRAIR-2125 Fab fragment variable domains, rendered at 6 Å resolution, in complex with SARS-CoV-2 RBD residues 470-494 (generated using PDB ID: 7N4L). The CDR H2, H3 and L3 loop are labelled in italics. The Fab heavy and light chain are colored yellow and purple and RBD residues are colored blue. The bottom panel shows a close-up view showing selected interactions formed between WRAIR-2125 residues and the SARS-CoV-2 RBD residues Y489 and F489. The unmodelled region of the CDR H3 loop is indicated with a dashed line. **(B)** As shown in A for 87G7.

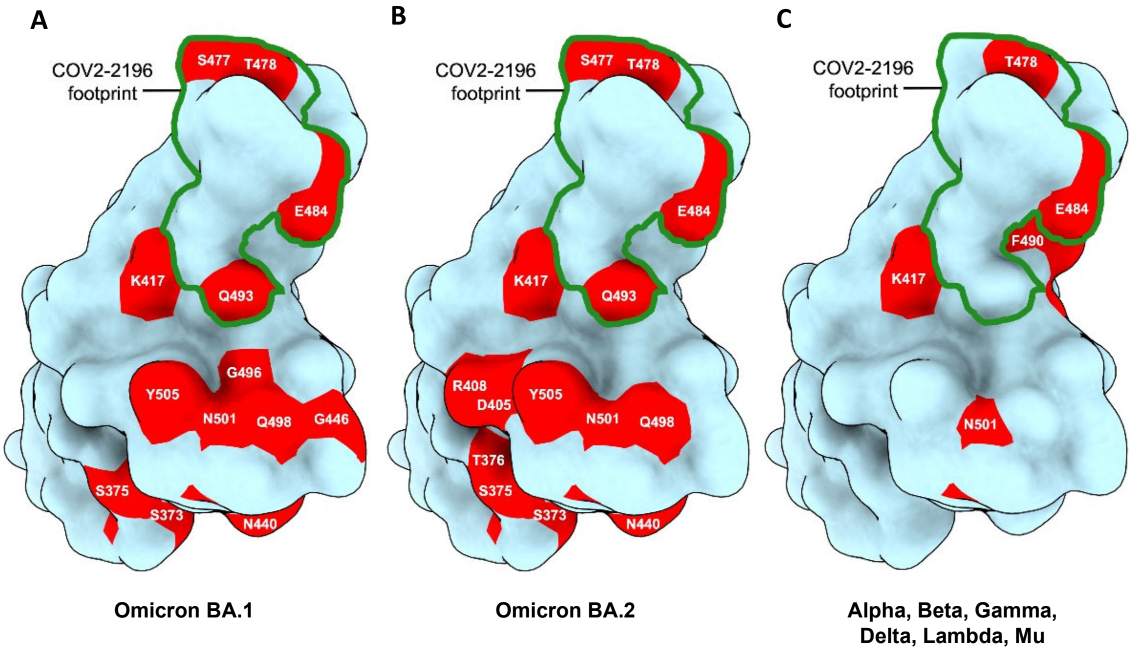

Figure S5. Location of VOC/VOI mutations relative to the COV2-2196 epitope. (A) Surface representation of the COV2-2196-bound SARS-CoV-2 S RBD (PDB ID: 7L7D), with mutations colored red that are found in Omicron BA.1 **(B)** Omicron BA.2 and **(C)** those present in Alpha, Beta, Gamma, Delta, Lambda or Mu. RBD residues within 4.5 Å of the modelled Fab fragment were considered to fall within the COV2-2196 footprint.

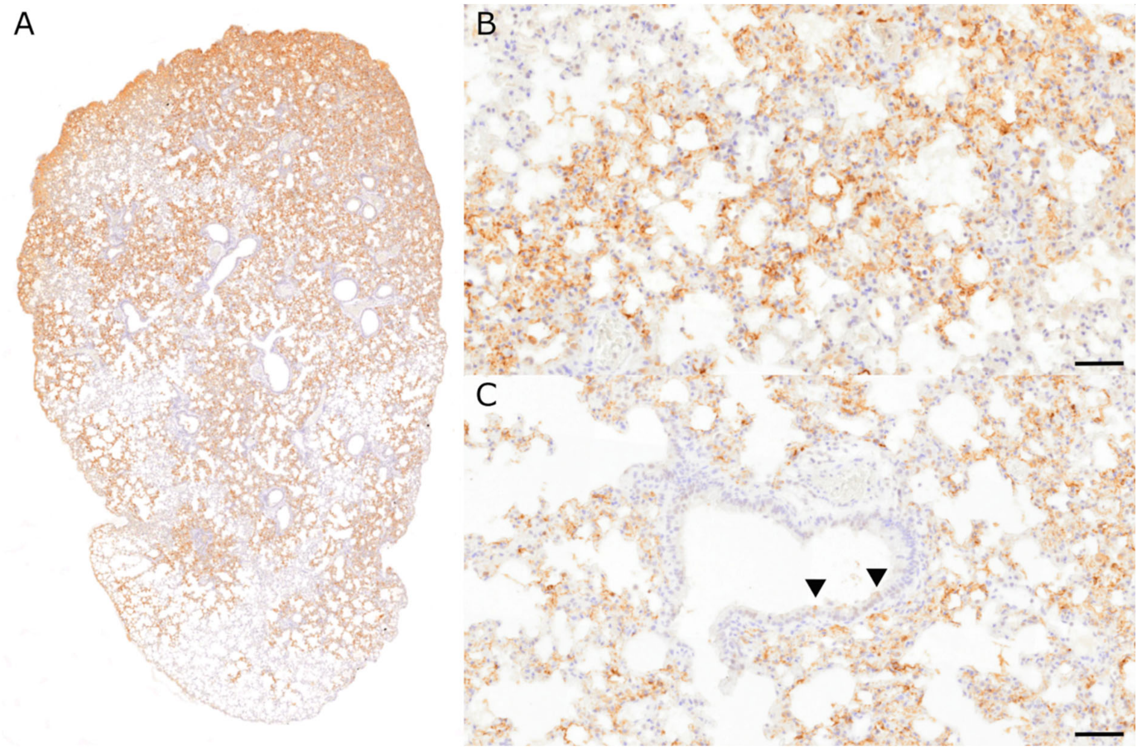

Figure S6. Representative images of SARS-CoV antigen immunohistochemistry in lungs of mice. (A) Overview of the left lung lobe showing abundant viral antigen (brown signal). **(B)** Higher magnification of alveoli, showing abundant viral antigen in pneumocytes. **(C)** Higher magnification of a bronchiole with single cells containing viral antigen (arrowheads). Scale bars: 50 µm. Related to Figure 4B and 4E.

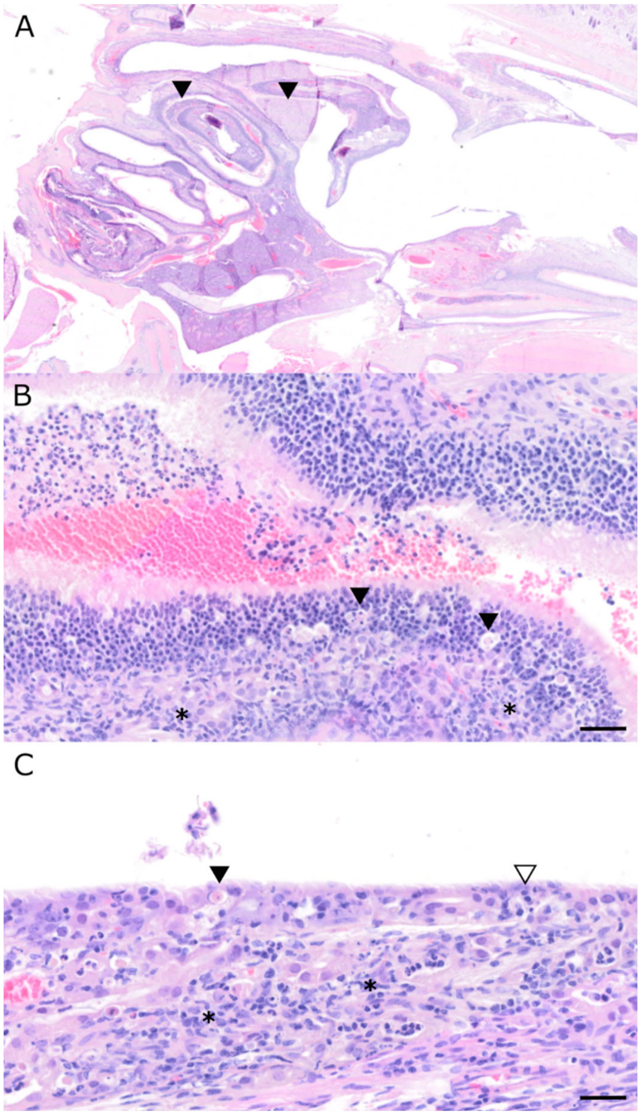

Figure S7. Representative images of SARS-CoV-2 induced histopathological lesions in the nasal turbinates of hamsters. **(A)** Overview of the nasal turbinates showing accumulation of intraluminal exudate (arrowheads). **(B)** Higher magnification of olfactory mucosa showing intraluminal cell debris admixed with erythrocytes, epithelial cell death (arrowheads) and subepithelial infiltrates of macrophages and neutrophils (asterisks) **(C)** Higher magnification of the respiratory epithelium showing epithelial disorganization, cell death (black arrowhead), intraepithelial (white arrowhead) and subepithelial (asterisks) inflammatory infiltrates, composed of macrophages and neutrophils. Scale bars: 50 µm. Related to Figure 5 B and E.

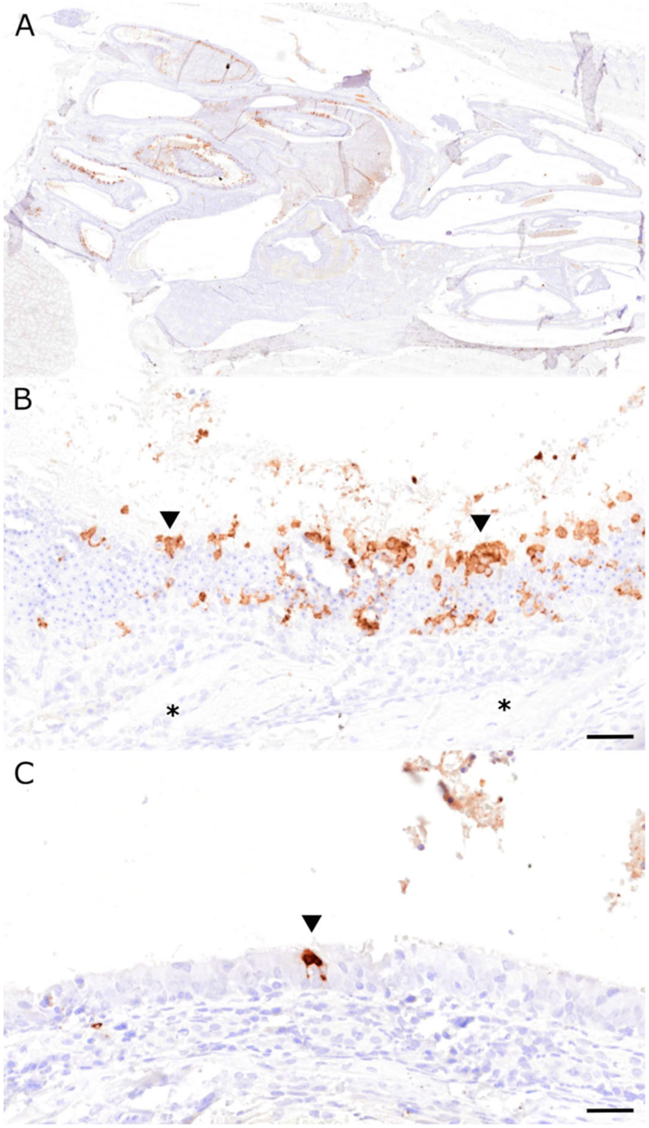

Figure S8. Representative images of SARS-CoV antigen immunohistochemistry in nasal turbinates of hamsters. (A) Overview of the nasal turbinates showing viral antigen (brown signal) in the epithelium and intraluminal exudate. **(B)** Higher magnification of olfactory mucosa showing viral antigen in many epithelial cells (arrowhead). **(C)** Higher magnification of the respiratory mucosa showing a single cell containing viral antigen (arrowhead). Scale bars: 50 µm. Related to Figure 5 C and F.

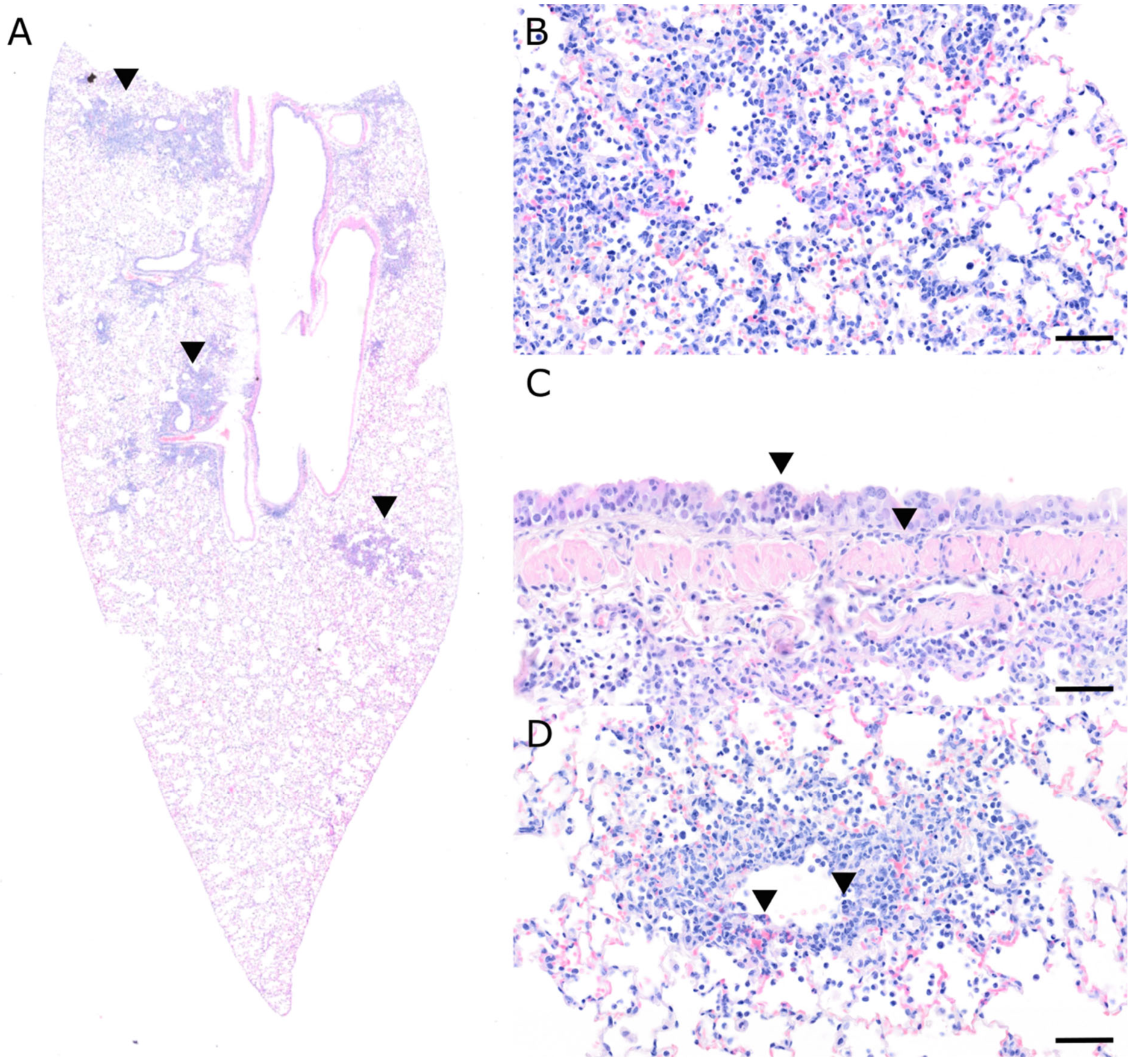

Figure S9. Representative images of SARS-CoV-2 induced histopathological lesions in the lung of hamsters. (A) Overview of the left lung lobe showing multifocal consolidated areas (arrowheads). **(B)** Higher magnification of alveoli showing intraluminal and septal infiltrates mainly composed of macrophages and neutrophils. **(C)** Higher magnification of a bronchus with intra- and subepithelial infiltrates (arrowheads), composed of lymphocytes and macrophages **(D)** Higher magnification of vessel with endothelialitis (arrowhead), mural and perivascular infiltrates composed of macrophages and neutrophils. Scale bars: 50 µm. Related to Figure 5 C and F.

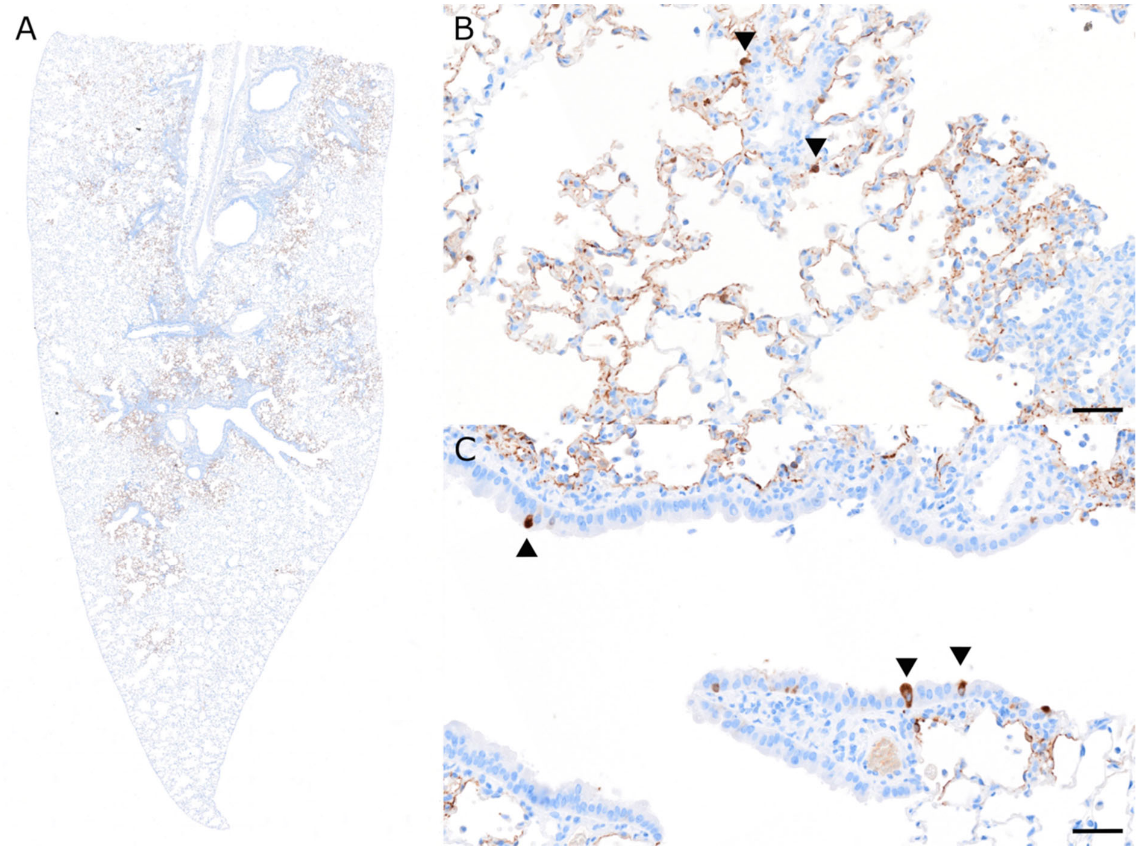

Figure S10. Representative images of SARS-CoV antigen immunohistochemistry in lungs of hamsters. (A) Overview of the left lung lobe showing abundant viral antigen (brown signal), concentrated in peribronchial areas. **(B)** Higher magnification of affected alveoli, showing abundant viral antigen in pneumocytes type 1 and single pneumocytes type II (arrowheads). **(C)** Higher magnification of a bronchus with single cells containing viral antigen (arrowheads). Scale bars: 50 µm. Related to Figure 5 B, C and E, F.

Table S1. Summary of data acquisition and image processing statistics. Related to Figure 2 and 3.

**Data Collection**		
Microscope	Titan Krios G4	
Voltage (keV)	300 keV	
Nominal magnification	130,000x	
Movie acquisition rate	~335 per hour	
Detector	Falcon 4	
Energy filer	Selectris-X	
Slit width (eV)	10	
Calibrated pixel size (Å)	0.929	
Cumulative exposure (e/Å^2)	51.5	
Dose rate (e/pixel/sec)	8.04	
Underfocus range (μm)	0.5 to 1.5	
Micrographs collected	1331 (0.02% FOM) + 2500 (0% FOM)	
**Reconstruction**	Global	Local
Final particles (no.)	133,550	72,118
Symmetry	C3	C1
B-factor (Å^2)	-84	-172
**Resolution (Å)**		
FSC 0.5 (masked)	3.3	5.9
FSC 0.143 (masked)	2.9	4.9
Resolution range (local)	2.6-11.1	4.7-7.9
**Refinement (composite)**		
Protein residues/atoms	3840/29907	
N-glycans/atoms	51/714	
**Resolution (Å)**		
FSC 0.5	3.5	
**Map correlation coefficient**		
Mask	0.63	
Box	0.68	
Volume	0.63	
Peaks	0.63	
**R.M.S. deviations**		
Bond Lengths (Å)	0.003	
Bond Angles (°)	0.713	
**MolProbity**		
Overall score	1.58	
Clashscore	4.76	
Ramachandran outliers (%)	0	
Ramachandran favoured (%)	95.24	
Rotamer outliers (%)	0	
C-beta outliers	0	
**EMRinger Score**	1.24	
**Privateer**		
Wrong anomer	0	
Wrong configuration	0	
Unphysical puckering amplitude	0	
In higher-energy conformations	0	

Table S2. Scoring system of mice lung SARS-CoV antigen immunohistochemistry. Related to Figure 4B and 4E.

**1. Immunohistochemistry**		
**1.1 Extent alveolar antigen**		
0	No	No specific signal
1	Minimal	Single small foci with immunolabelled pneumocytes, less than 1 % of alveoli
2	Mild	Multifocal areas with immunolabelled pneumocytes, 2-25 % of alveoli
3	Moderate	Multifocal areas with immunolabelled pneumocytes, 26-50 % of alveoli
4	Marked	Multifocal areas with immunolabelled pneumocytes, 51-75 % of alveoli
5	Subtotal	Multifocal areas with immunolabelled pneumocytes, > 75 % of alveoli
**1.2 Extent airway (bronchi and bronchioli) antigen**		
0	No	No specific signal
1	Minimal	Single small foci with immunolabelled epithelia, less than 1 % of airways
2	Mild	Multifocal areas with immunolabelled epithelia, 2-25 % of airways
3	Moderate	Multifocal areas with immunolabelled epithelia, 26-50 % of airways
4	Marked	Multifocal areas with immunolabelled epithelia, 51-75 % of airways
5	Subtotal	Multifocal areas with immunolabelled epithelia, > 75 % of airways
Total immunohistochemistry score		
**Semiquantitative score (antigen):** Sum of alveolar and airway score		

Table S3. Scoring system of hamster lung (alveolar, airway and vascular) lesions, Related to Figure 5B and 5E.

**1. Alveolar lesions**		
**1.1 Extent alveolar inflammation**		
0	No	No inflammation
1	Minimal	Single small foci of mild inflammatory infiltrates, affecting max. 1% of tissue, (significance questionable)
2	Mild	2-25 % of tissue affected
3	Moderate	26-50 % of tissue affected
4	Marked	51-75 % of tissue affected
5	Subtotal	> 75 % of tissue affected
**1.2 Severity alveolar inflammation (scored in area of maximal severity)**		
0	No	No inflammatory infiltrates
1	Minimal	Few inflammatory cells in alveolar septae or lumina, alveolar architecture maintained (significance questionable)
2	Mild	Mild septal and luminal infiltrates (2-3 cells thick), alveolar architecture maintained
3	Moderate	Moderate septal and luminal infiltrates, occasionally obscuring alveolar architecture
4	Marked	Marked septal and luminal infiltrates with large areas with completely obscured alveolar architecture
**1.3 Alveolar damage**		
0/1	No/Yes	Necrosis/desquamation/loss (denuded septae) of alveolar cells
0/1	No/Yes	Intraalveolar fibrin/hyaline membranes
0/1	No/Yes	Atypical/large or multinucleated cells lining alveoli
0/1	No/Yes	Alveolar edema
0/1	No/Yes	Alveolar hemorrhage
**Total alveolar lesions score**		
**Sum of all scores**		
**2. Airway lesions**		
**2.1 Extent airway inflammation**		
0	No	No inflammation
1	Minimal	Single small foci of mild inflammatory infiltrates, affecting max. 1% of tissue, (significance questionable)
2	Mild	2-25 % of tissue affected
3	Moderate	26-50 % of tissue affected
4	Marked	51-75 % of tissue affected
5	Subtotal	> 75 % of tissue affected
**2.2 Severity airway (bronchi/bronchiole) inflammation (scored in area of maximal severity)**		
0	No	No inflammation
1	Minimal	Rare peribronchial/peribronchiolar, mild, mononuclear infiltrates (significance questionable)
2	Mild	Mild, mononuclear and granulocytic bronchitis/bronchiolitis (with exocytosis of inflammatory cells into epithelium, occasional single cell necrosis and mild, peribronchial/peribronchiolar infiltrates)
3	Moderate	Moderate, mononuclear and granulocytic to necrotizing bronchitis/bronchiolitis (with exocytosis of inflammatory cells into epithelium, frequent single cell necrosis and intraluminal debris + moderate, peribronchial/peribronchiolar infiltrates)
4	Marked	Marked, mononuclear and granulocytic and necrotizing bronchitis/bronchiolitis with widespread exocytosis of inflammatory cells into epithelium, frequent epithelial necrosis and intraluminal debris, severe peribronchial/peribronchiolar infiltrates)
**Total airway lesions score**		
**Sum of all scores**		

**3. Vascular lesions**		
**3.1 Extent vaculopathy/vasculitis (large and medium sized vessels with visible tunica media, characterized by endothelialitis and mural infiltrates with disruption of vessel walls)**		
0	No	No lesions
1	Minimal	Single vessels with mild inflammation (significance questionable)
2	Mild	2-25 % of vessels affected
3	Moderate	26-50 % of vessels affected
4	Marked	51-75 % of vessels affected
5	Subtotal	> 75 % of vessels affected
**3.2 Severity perivascular infiltrates (scored in area of maximal severity)**		
0	No	No inflammation
1	Minimal	Single vessels with endothelial hypertrophy and few perivascular cells (no continuous cuff, significance questionable)
2	Mild	1-2 cell layers of perivascular cuffs
3	Moderate	3-5 cell layers of perivascular cuffs
4	Marked	> 5 cell layers of perivascular cuffs
**3.3 Vascular lesions**		
0/1	No/Yes	Perivascular edema
0/1	No/Yes	Perivascular hemorrhage
**Total vascular lesions score**		
Sum of all scores		

Semiquantitative score (lesion lung): sum of total alveolar, airway and vascular lesions score

Table S4: Scoring system of nasal cavity lesions. Related to Figure 5B and 5E.

**1. Nose lesions**		
**1.1 Inflammation respiratory epithelium :**		
0	No	No inflammation
1	Minimal	occasional foci with few inflammatory cells, less than 1% of tissue affected
2	Mild	2-25% of tissue affected
3	Moderate	26-50% of tissue affected
4	Marked	51-75% of tissue affected
5	Subtotal	> 75 % of tissue affected
**1.2 Necrosis of respiratory epithelium :**		
0	No	no necrosis
1	Minimal	occasional foci of few necrotic cells, less than 1% of tissue affected
2	Mild	2-25% of tissue affected
3	Moderate	26-50% of tissue affected
4	Marked	51-75% of tissue affected
5	Subtotal	> 75 % of tissue affected
**1.3 Epithelial hyper-and/or metaplasia respiratory epithelium**		
0/1	No/Yes	
**1.4 Combined score respiratory epithelium**		
	Sum of 1.1 to 1.3	
**1.5 Inflammation olfactory epithelium**		
0	No	No inflammation
1	Minimal	occasional foci with few inflammatory cells, less than 1% of tissue affected
2	Mild	2-25% of tissue affected
3	Moderate	26-50% of tissue affected
4	Marked	51-75% of tissue affected
5	Subtotal	> 75 % of tissue affected
**1.6 Necrosis olfactory epithelium**		
0	No	no necrosis
1	Minimal	occasional foci of few necrotic cells, less than 1% of tissue affected
2	Mild	2-25% of tissue affected
3	Moderate	26-50% of tissue affected
4	Marked	51-75% of tissue affected
5	Subtotal	> 75 % of tissue affected
**1.7 Combined score olfactory epithelium**		
	Sum of 1.5 and 1.6	
**1.8 Intraluminal exudate**		
0/1	No/Yes	
**1.9 Vasculopathy (ural infiltrates , endothelial hypertrophy)**		
0/1	No/Yes	
**1.10 Combined Score nose**		
**Semiquantitative score (lesion):** Sum of 1.4 and 1.7-1.9		

Table S5: Scoring system of hamster lung and nose SARS-CoV-2 antigen immunohistochemistry. Related to Figure 5C and 5F.

**1. Immunohistochemistry (lung)**		
**1.1 Extent alveolar antigen**		
0	No	No specific signal
1	Minimal	Single small foci with immunolabelled pneumocytes, less than 1 % of alveoli
2	Mild	Multifocal areas with immunolabelled pneumocytes, 2-25 % of alveoli
3	Moderate	Multifocal areas with immunolabelled pneumocytes, 26-50 % of alveoli
4	Marked	Multifocal areas with immunolabelled pneumocytes, 51-75 % of alveoli
5	Subtotal	Multifocal areas with immunolabelled pneumocytes, > 75 % of alveoli
**1.2 Extent airway (bronchi and bronchioli) antigen**		
0	No	No specific signal
1	Minimal	Single small foci with immunolabelled epithelia, less than 1 % of airways
2	Mild	Multifocal areas with immunolabelled epithelia, 2-25 % of airways
3	Moderate	Multifocal areas with immunolabelled epithelia, 26-50 % of airways
4	Marked	Multifocal areas with immunolabelled epithelia, 51-75 % of airways
5	Subtotal	Multifocal areas with immunolabelled epithelia, > 75 % of airways
**Total immunohistochemistry score**		
**Semiquantitative score (antigen):** Sum of alveolar and airway score		
**2. Immunohistochemistry nose**		
**2.1 Respiratory Epithelium:**		
0	No	No signal
1	Minimal	single positive cells, up to 1% of epithelium
2	Mild	2-25% of epithelium positive
3	Moderate	26-50% of epithelium positive
4	Marked	51-75% of epithelium positive
5	Subtotal	> 75 % of epithelium positive
**2.2 Olfactory Epithelium:**		
0	No	No signal
1	Minimal	single positive cells, up to 1% of epithelium
2	Mild	2-25% of epithelium positive
3	Moderate	26-50% of epithelium positive
4	Marked	51-75% of epithelium positive
5	Subtotal	> 75 % of epithelium positive
**2.3 Intraluminal exudate**		
0/1	No/Yes	
**2.4 Subepithelial cells**		
0/1	No/Yes	
**2.5 Combined Score**		
**Semiquantitative score (antigen):** Sum of all scores		
